# Supplementary material for: Multi-omics approaches define novel aphid effector candidates associated with virulence and avirulence phenotypes
Source: BMC Genomics. 2024 Nov 11;25:1065. doi: 10.1186/s12864-024-10984-x (PMC11552303; doi:10.1186/s12864-024-10984-x)
Supplement: Supplementary file 2 — Supplementary Material 2. [file 12864_2024_10984_MOESM2_ESM.pptx]

## Slide 1
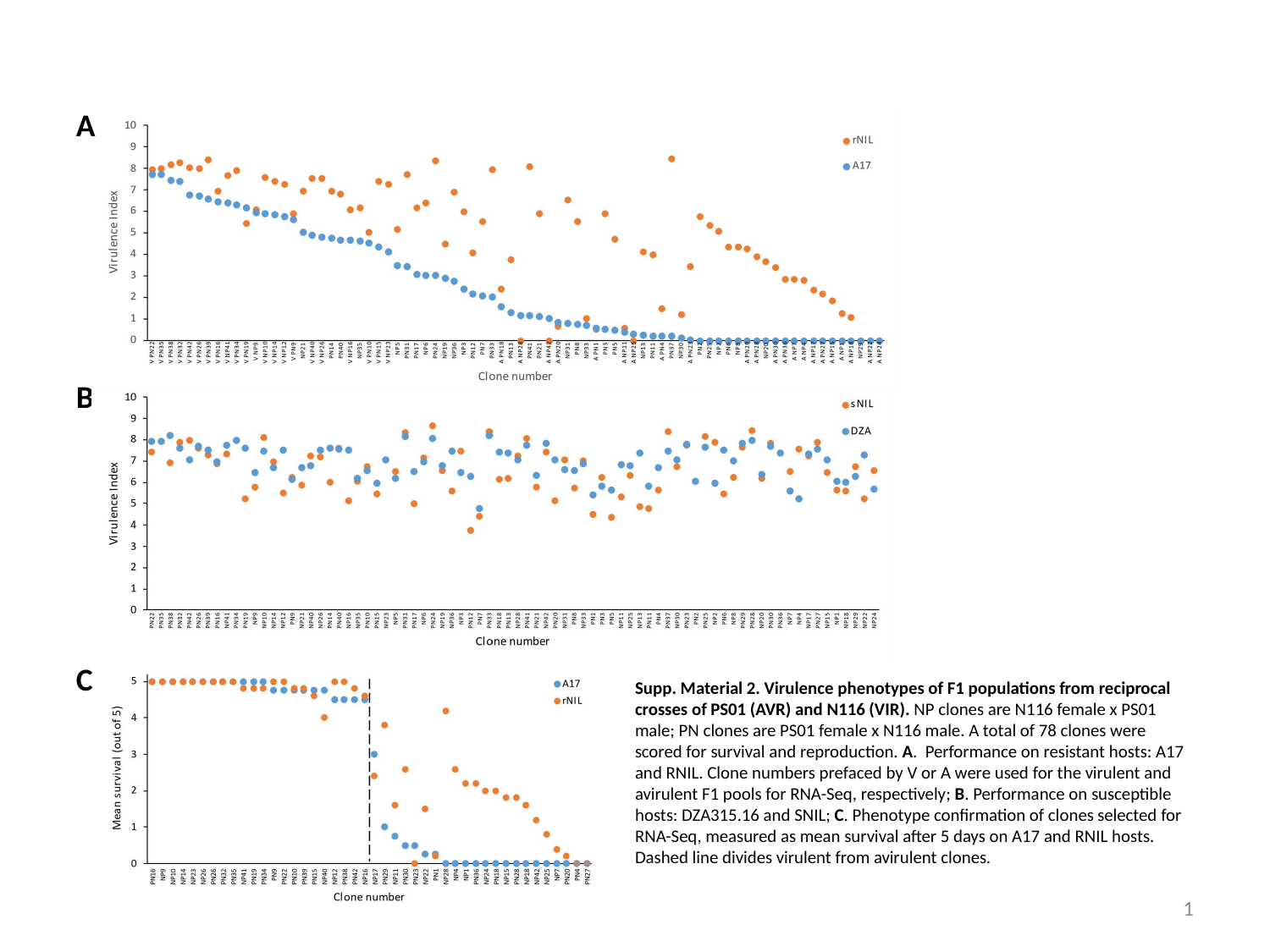

A
B
C
Supp. Material 2. Virulence phenotypes of F1 populations from reciprocal crosses of PS01 (AVR) and N116 (VIR). NP clones are N116 female x PS01 male; PN clones are PS01 female x N116 male. A total of 78 clones were scored for survival and reproduction. A. Performance on resistant hosts: A17 and RNIL. Clone numbers prefaced by V or A were used for the virulent and avirulent F1 pools for RNA-Seq, respectively; B. Performance on susceptible hosts: DZA315.16 and SNIL; C. Phenotype confirmation of clones selected for RNA-Seq, measured as mean survival after 5 days on A17 and RNIL hosts. Dashed line divides virulent from avirulent clones.
1
